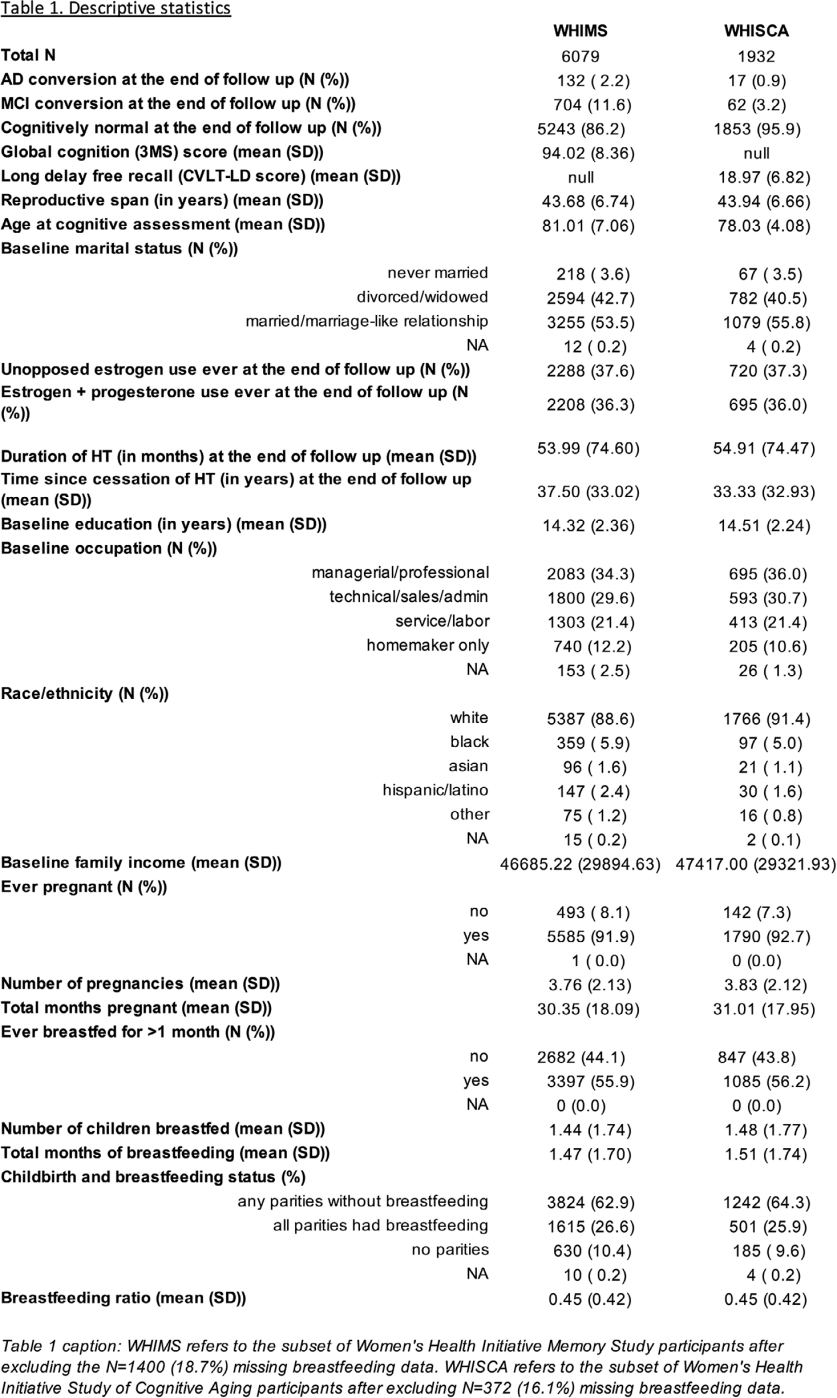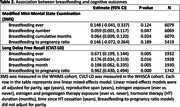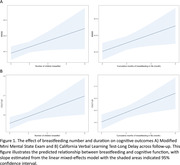# Women’s history of breastfeeding is positively associated with post‐menopausal cognitive function

**DOI:** 10.1002/alz.091063

**Published:** 2025-01-09

**Authors:** Molly Fox, Jennifer E. Bramen, Dayoon Kwon, Sonel Raj, Marcus Chang, Carolyn Crandall, Verna R. Porter, Mark A. Espeland, Prabha Siddarth

**Affiliations:** ^1^ UCLA, Los Angeles, CA USA; ^2^ Saint John’s Cancer Institute at Providence Saint John’s Health Center, Santa Monica, CA USA; ^3^ UCLA Fielding School of Public Health, Los Angeles, CA USA; ^4^ UCLA David Geffen School of Medicine, Los Angeles, CA USA; ^5^ Pacific Brain Health Center, Pacific Neuroscience Institute Foundation, Santa Monica, CA USA; ^6^ Wake Forest University School of Medicine, Winston Salem, NC USA; ^7^ David Geffen School of Medicine at University of California Los Angeles, Los Angeles, CA USA

## Abstract

**Background:**

Women’s reproductive experiences may enact reorganization of physiological systems with lifelong health consequences. We test the hypothesis that women’s history of breastfeeding will be positively associated with neurocognitive benefits in post‐menopausal women. This hypothesis is justified by breastfeeding’s well‐established benefits for mothers’ glucose homeostasis, beta‐cell function, adipose tissue mobilization, and lipid metabolism, which would plausibly be beneficial for later‐life brain health.

**Method:**

The Women’s Health Initiative (WHI) was a long‐term, large, US health study in the 1990s‐early 2000s. The WHI Memory Study (WHIMS) was an ancillary study in which cognitively healthy women at baseline were annually assessed. A subset of WHIMS participants were recruited into the WHI Study of Cognitive Aging (WHISCA), which included more comprehensive annual assessments of cognitive function and mood. We use WHIMS participant scores on the Modified Mini Mental State Exam “3MS,” which measures global cognitive functioning, and WHISCA participant scores on the California Verbal Learning Test‐Long Delay (CVLT‐LD), which measures long‐term memory. We employed linear mixed‐effects models to examine the association of 3MS and CVLT‐LD scores with women’s breastfeeding history, controlling for the effects of parity, age, reproductive span, hormone therapies (HT) and duration, and time since HT cessation.

**Result:**

In the WHIMS (N = 6,069) and WHISCA (N = 1,932) cohorts (Table 1 demographics), we found that the number of children a woman breastfed was positively associated with better global cognition (b = 0.059, p = 0.047) and long‐term memory (b = 0.176, p = 0.016). The cumulative number of months a woman breastfed was positively associated with better global cognition (b = 0.064, p = 0.024) and long‐term memory (b = 0.198, p = 0.005). Women who had breastfed ever for at least one month, compared to those who did not, exhibited better long‐term memory (b = 0.671, p = 0.005), and no significant effect for global cognition. Women with a higher breastfeeding‐to‐pregnancy duration ratio exhibited better long‐term memory (b = 0.962, p = 0.000), with no significant effect for global cognition (Table 2, Fig. 1).

**Conclusion:**

Our findings indicate long‐term cognitive benefits of breastfeeding for women, above and beyond any effects of parity. It is possible that breastfeeding could be beneficial for women by endowing resilience against neurodegenerative disorders, consistent with our previous observations from two small cohort pilot studies.